# Psychosocial and Environmental Correlates of Walking, Cycling, Public Transport and Passive Transport to Various Destinations in Flemish Older Adolescents

**DOI:** 10.1371/journal.pone.0147128

**Published:** 2016-01-19

**Authors:** Hannah Verhoeven, Dorien Simons, Delfien Van Dyck, Jelle Van Cauwenberg, Peter Clarys, Ilse De Bourdeaudhuij, Bas de Geus, Corneel Vandelanotte, Benedicte Deforche

**Affiliations:** 1 Department of Public Health, Faculty of Medicine and Health Sciences, Ghent University, De Pintelaan 185, B-9000 Ghent, Belgium; 2 Department of Movement and Sport Sciences, Faculty of Physical Education and Physical Therapy, Vrije Universiteit Brussel, Pleinlaan 2, B-1050 Brussels, Belgium; 3 Fund for Scientific Research Flanders (FWO), Egmontstraat 5, B-1000 Brussels, Belgium; 4 Department of Movement and Sport Sciences, Faculty of Medicine and Health Sciences, Ghent University, Watersportlaan 2, B-9000 Ghent, Belgium; 5 Department of Human Physiology, Faculty of Physical Education and Physical Therapy, Vrije Universiteit Brussel, Pleinlaan 2, B-1050 Brussels, Belgium; 6 Central Queensland University, Institute for Health and Social Science Research, Physical Activity Research Group, Bruce Highway, Rockhampton QLD 4702, Australia; Old Dominion University, UNITED STATES

## Abstract

**Background:**

Active transport is a convenient way to incorporate physical activity in adolescents’ daily life. The present study aimed to investigate which psychosocial and environmental factors are associated with walking, cycling, public transport (train, tram, bus, metro) and passive transport (car, motorcycle, moped) over short distances (maximum eight kilometres) among older adolescents (17–18 years), to school and to other destinations.

**Methods:**

562 older adolescents completed an online questionnaire assessing socio-demographic variables, psychosocial variables, environmental variables and transport to school/other destinations. Zero-inflated negative binomial regression models were performed.

**Results:**

More social modelling and a higher residential density were positively associated with walking to school and walking to other destinations, respectively. Regarding cycling, higher self-efficacy and a higher social norm were positively associated with cycling to school and to other destinations. Regarding public transport, a higher social norm, more social modelling of siblings and/or friends, more social support and a higher land use mix access were positively related to public transport to school and to other destinations, whereas a greater distance to school only related positively to public transport to school. Regarding passive transport, more social support and more perceived benefits were positively associated with passive transport to school and to other destinations. Perceiving less walking and cycling facilities at school was positively related to passive transport to school only, and more social modelling was positively related to passive transport to other destinations.

**Conclusions:**

Overall, psychosocial variables seemed to be more important than environmental variables across the four transport modes. Social norm, social modelling and social support were the most consistent psychosocial factors which indicates that it is important to target both older adolescents and their social environment in interventions promoting active transport. Walking or cycling together with siblings or friends has the potential to increase social norm, social modelling and social support towards active transport.

## Introduction

Sufficient physical activity is associated with numerous health benefits such as the prevention of overweight and obesity, developing a healthy cardiovascular system, and a long-term protective effect on bone health and even on mental health [1,2]. Despite these health benefits, about 81% of adolescents worldwide do not meet the physical activity guideline of 60 minutes of moderate- to vigorous-intensity physical activity a day [3]. Furthermore, there is a steep decline in physical activity levels during adolescence and this decline continues into young adulthood [4,5,6]. Active transport (e.g. walking and cycling) is a convenient way to incorporate physical activity into adolescents’ daily activities and increase overall physical activity levels [5,7,8,9]. Furthermore, public transport might be another opportunity to accumulate sufficient physical activity since use of public transport generally involves some walking or cycling [10,11]. However, in Europe, adolescents can obtain a regular driving license from the age of 18. Previous studies showed that obtaining a driving license is related to a decline in walking and cycling for transport [12,13,14]. Furthermore, the transition from late adolescence to young adulthood has been shown to be a critical period characterized by major life changes (i.e. beginning paid work or starting higher education) that are associated with a decrease in physical activity [15,16,17]. Therefore, it is important to promote active transport at the age of 17–18 years (the so-called older adolescents), just before this transitional period in which habitual car driving patterns are established [14].

Previous studies mainly focused on active transport to school [18,19,20,21]. Distance was mentioned as one of the most consistent predictors of active transport in several studies among adolescents, with those adolescents living closer to school being more likely to commute actively [18,19,20,22]. However, even for destinations within a feasible walking or cycling distance, older adolescents regularly use passive transport modes such as a car, motorcycle or moped [20]. In the study by Van Dyck et al. [21], feasible distances for active transport to school in Belgian older adolescents were found to be two kilometres for walking and eight kilometres for cycling. In that sample, 42% commuted passively to school (including public transport) although 44% of these passive commuters lived within a feasible walking (two km) or cycling (eight km) distance from school [21]. This indicates that there is room to increase active transport to school in older adolescents, and possibly also to other destinations. While adolescents may not always live within a feasible active commuting distance from school, multiple other destinations are much more likely to be within walking or cycling distance. Therefore, it is of importance to investigate correlates of active transport within a feasible distance, both to school and other destinations.

In order to develop effective intervention programs to promote active transport in older adolescents, it is important to identify correlates of active transport in this target group. Ecological models state that health behaviours such as active transport are influenced by various factors at multiple levels, including psychosocial and environmental factors [23]. Previous research investigating correlates of active transport in adolescents mainly focused on young adolescents (12–16 years) living outside Europe [18,19,24,25,26]. However, correlates of active transport can vary among adolescents of different ages and countries. Compared to the US, Europe (especially Northern and Western Europe) is a cycle-friendly continent. Furthermore, research on correlates of active transport specifically in older adolescents is very limited. A qualitative study among Belgian older adolescents, using focus groups, indicated that high autonomy, good social support, short travel time and low cost facilitates choosing active travel modes over other transport forms [27]. In that study, it was also indicated that it is important to have access to appropriate cycling facilities (e.g. good bicycle storage) at the destination. Factors that did not seem to have a large influence on their choice of transport mode were safety from traffic and crime, ecology (influence of walking/cycling versus car on the environment) and physical health (physical fitness, inhaling car exhaust). To our knowledge, only two quantitative studies investigated correlates of active transport in European 17–18 year olds [21,28]. In a study by Van Dyck et al. [21], perceiving higher neighbourhood walkability and more social modelling had a positive association with active transport to school. Social norm, social support, walking and cycling infrastructure, and traffic safety were not associated with active transport to school [21]. In a study by Deforche et al. [28], higher self-efficacy, modelling of family and social support of family and friends were related to higher levels of total active transport (to school and other destinations in leisure time). Furthermore, higher land use mix diversity, higher street connectivity and more aesthetically pleasing neighbourhoods were also positively related to total active transport. While these two studies measured active transport in older adolescents, they assessed the psychosocial correlates regarding physical activity in general (e.g. self-efficacy for general physical activity rather than for cycling for transport). However, it is likely that correlates of walking or cycling for transport differ from those of general physical activity [29].

In order to promote active transport for short distance travel in older adolescents, evidence on reasons for choosing other transport modes might give us important insights. Public transport (train, tram, bus, metro) and passive transport (car, motorcycle, moped) are commonly used transport modes within this age group [21,30]. Yet, evidence on correlates of public and passive transport use in (older) adolescents is lacking. In this context it is important to note that public transport should not be listed as a passive transport mode since use of public transport generally involves some walking or cycling [10,11].

In summary, there is a lack of research on correlates of different transport modes for short distance travel (maximum eight kilometres) to various destinations in older adolescents. Consequently, the purpose of this study was to investigate which psychosocial and environmental factors are associated with walking, cycling, public transport and passive transport over short distances in Flemish older adolescents, not only to school but also to other destinations.

## Methods

### Participants and protocol

Participants were recruited from randomly selected secondary schools across Flanders (convenience sampling; n = 25). An email was sent to principals, coordinators or study counsellors of the secondary schools with an invitation to participate, and this was followed-up by a phone call. After agreement of schools to participate, each school’s contact person ensured that a link to an online questionnaire reached pupils of the final two years of secondary school who could voluntarily and anonymously participate in the study. Nine out of 25 contacted secondary schools agreed to participate (response rate = 36.0%), accounting for a total of 2046 pupils in the last two years of secondary school. In addition, social media (such as Facebook) were used as a channel to recruit participants. Social networking websites seem to be an effective strategy to recruit participants within this age group [31]. A total of 1145 older adolescents started the questionnaire, of whom 613 completed the questionnaire entirely.

Participants were informed that consent was automatically obtained when they voluntarily completed the questionnaire. Informed consent of parents or guardians was not obtained since parental consent is not required from the age of 16 when questions are not related to a sensitive topic [32,33] and information is related to a non-identified or non-identifiable natural person [34]. The study protocol was approved by the medical ethical committee of the Vrije Universiteit Brussel referring to The Privacy Act of December 8th, 2012 on the protection of privacy in relation to the processing of personal data [34].

### Measurements

In this cross-sectional study, participants were asked to complete a self-reported online questionnaire assessing socio-demographic variables, general transport data, transport to school and to other destinations, psychosocial variables and environmental variables. Data collection occurred during March-May 2013. The questionnaire consisted of questions derived from validated questionnaires [35,36,37,38,39] and was adjusted to better fit the target group according to the results of a prior exploratory qualitative study using focus groups [27].

#### Socio-demographic variables

Self-reported socio-demographic variables included gender, age, nationality (Belgian, other) and grade (penultimate year of secondary school, last year of secondary school). Furthermore, living situation was dichotomized into living with (grand)parents (coded 0) and living with partner/alone/other (coded 1), whereas living environment was dichotomized into rural area (countryside/village, coded 0) and urban area (suburban area/city, coded 1). Education of mother and education of father were combined into one variable representing socio-economic status (SES) with low SES (no parent with a Bachelor’s degree or higher, coded 0) and high SES (at least one parent with a Bachelor’s degree or higher, coded 1). Finally, educational type was dichotomized into occupational studies (coded 0) and general/technical studies (coded 1). Participants also reported their height and weight, which were used to calculate Body Mass Index (BMI).

#### General transport data

General transport data included possession of a driving license for a car; ownership of moped, car/motorcycle and bicycle; sharing and/or borrowing capability of moped, car/motorcycle and bicycle; and pass ownership for public transport and for bicycle sharing schemes.

#### Transport to school and to other destinations

To assess transport to school and to other destinations, questions derived from the validated International Physical Activity Questionnaire (IPAQ) [35,40] were used. Participants were asked about frequency (days/week) and average daily duration of trips with different transport modes within the last seven days. By multiplying frequency and duration of trips, eight dependent variables were obtained: minutes/week walking, cycling, public transport use and passive transport use (car/motorcycle/moped) to school, and minutes/week walking, cycling, public transport use and passive transport use (car/motorcycle/moped) to other destinations.

#### Psychosocial variables

This part of the questionnaire was based on an existing questionnaire [36], that was adjusted to the specific target group according to the results of a prior explorative qualitative study [27]. The following psychosocial variables were assessed: self-efficacy, social norm, social modelling, social support, perceived benefits and perceived barriers. Self-efficacy was assessed by asking participants how confident they were to choose active transport over other transport modes in 11 potentially difficult situations (i.e. bad weather, dark, when tired). Social norm was measured by asking if participants believed that significant others wanted them to (a) walk or cycle; (b) take a car/motorcycle/moped; (c) use public transport. Modelling was assessed by asking how frequently significant others (a) walk or cycle; (b) take a car/motorcycle/moped; (c) use public transport. To investigate social support, participants were asked how often significant others encourage them to (a) walk or cycle; (b) take a car/motorcycle/moped; (c) use public transport and how often they do this together with them. To measure perceived benefits, participants were asked about potential benefits (i.e. health, cost, independence) of (a) walking or cycling; (b) taking the car/motorcycle/moped; (c) using public transport. Perceived barriers were assessed by asking participants about potential barriers (i.e. time, accidents, delays) of (a) walking or cycling; (b) taking the car/motorcycle/moped; (c) using public transport. A summary of the measures of psychosocial variables is shown in Table 1. Averages of item scores were used for data analyses.

**Table 1:** Summary of measures and mean scores (SD) of psychosocial and environmental factors.

Scale (composition)		Response category	α	M (SD)
**Psychosocial**				
Self-efficacy				
*walking/cycling*	11 items	five-point scale^a^	0.92	3.47 (1.05)
Social norm				
*walking/cycling*	partner; parents;	five-point scale^b^	0.89	3.01 (1.22)
	brothers/sisters; friends			
*public transport*	partner; parents;	five-point scale^b^	0.91	2.72 (1.15)
	brothers/sisters; friends			
*passive transport*	partner; parents;	five-point scale^b^	0.93	2.24 (1.14)
	brothers/sisters; friends			
Social modelling				
*walking/cycling*	partner; parents;	five-point scale^c^	0.75	3.35 (1.07)
	brothers/sisters; friends			
*public transport*	partner;	five-point scale^c^	0.52	1.63 (2.99);
	parents;			1.19 (1.68);
	brothers/sisters;			1.56 (2.70);
	friends			1.26 (3.66)
*passive transport*	partner; parents;	five-point scale^c^	0.82	3.54 (1.12)
	brothers/sisters; friends			
Social support				
*walking/cycling*	partner; parents;	five-point scale^d^	0.83	2.62 (0.84)
	brothers/sisters; friends			
*public transport*	partner; parents;	five-point scale^d^	0.87	2.28 (0.86)
	brothers/sisters; friends			
*passive transport*	partner; parents;	five-point scale^d^	0.87	2.44 (0.88)
	brothers/sisters; friends			
Perceived benefits				
*walking/cycling*	18 items	five-point scale^b^	0.93	3.59 (0.85)
*public transport*	6 items	five-point scale^b^	0.80	2.90 (0.93)
*passive transport*	7 items	five-point scale^b^	0.87	3.19 (0.96)
Perceived barriers				
*walking/cycling*	22 items	five-point scale^d^	0.94	2.17 (0.80)
*public transport*	8 items	five-point scale^d^	0.87	2.67 (0.91)
*passive transport*	11 items	five-point scale^d^	0.87	2.60 (0.97)
**Environmental**				
Residential density	3 items	five-point scale^e^		2.50 (1.03)
Land use mix diversity	8 items	five-point scale^f^		3.48 (0.91)
Land use mix access	6 items	four-point scale^g^		2.89 (0.54)
Street connectivity	5 items	four-point scale^g^		2.60 (0.54)
Walking and cycling facilities	12 items	four-point scale^g^		2.54 (0.48)
Aesthetics	4 items	four-point scale^g^		2.73 (0.58)
Perceived safety from traffic	8 items	four-point scale^g^		2.56 (0.36)
Perceived safety from crime	6 items	four-point scale^g^		3.10 (0.72)
Facilities at school	5 items	two-point scale^h^		0.51 (0.21)

^a^ five-point scale from 1 (know I cannot do it) to 5 (know I can do it)

^b^ five-point scale from 1 (strongly disagree) to 5 (strongly agree)

^c^ five-point scale: never or once per year, 1 time per month, several times per month, several times per week, almost every day

^d^ five-point scale from 1 (never) to 5 (always)

^e^ five-point scale from 1 (none) to 5 (all)

^f^ five-point scale: 1–5, 6–10, 11–20, 20–30, > 30 minutes

^g^ four-point scale: strongly disagree, somewhat disagree, somewhat agree, strongly agree

^h^ two-point scale: 1 (yes) and 2 (no).

#### Perceived environmental variables

Perceived environmental variables were assessed using questions derived from validated questionnaires: the European environmental questionnaire (ALPHA questionnaire) [37] and the Neighbourhood Environment Walkability Scale (NEWS) [38,39]. ‘Neighbourhood’ was defined as ‘the environment within a walking or cycling distance of 10–15 minutes from home’. Data were cleaned and analysed conform the ALPHA environmental questionnaire Manual [41] and the NEWS scoring procedures [42]. The following perceived environmental variables were assessed: residential density, land use mix diversity, land use mix access, street connectivity, walking and cycling facilities, aesthetics, safety from traffic and safety from crime. Furthermore, facilities at school and self-reported distance to school (in kilometres) was assessed. A summary of the measures of environmental variables is shown in Table 1.

### Data analyses

Data were analysed using R Studio version 3.1.0 (see S1 Dataset). To investigate the associations of psychosocial and environmental factors with walking, cycling, public transport and passive transport, zero-inflated negative binomial (ZINB) regression models were used. ZINB models were used since the dependent variables were positively skewed and contained a large number of zero counts. Vuong tests supported the need to use zero-inflated regression models [43] and Akaike’s Information Criterium showed that a ZINB model was preferred over a zero-inflated poisson model. ZINB models evaluate the relationships with the odds of non-participation in walking, cycling, public transport and passive transport to school and to other destinations. Simultaneously, among those who did make use of these transport modes in the last week, ZINB models evaluate the relationships with weekly minutes engaged in these transport modes. Hence, one ZINB model might yield two regression coefficients for each independent variable: an odds ratio (OR) (for the relationship between the independent variable and the odds of not engaging in walking, cycling, public transport or passive transport) and a negative-binomial model regression coefficient (representing the proportional changes in minutes/week walking, cycling, public transport or passive transport with a one-unit increase in the independent variable for those participants who did engage in walking, cycling, public transport or passive transport).

In a first step, ZINB models with socio-demographic variables (gender, age, BMI, SES, education) were built for walking to school and walking to other destinations, cycling to school and cycling to other destinations, public transport to school and public transport to other destinations, and passive transport to school and passive transport to other destinations (eight models). Furthermore, the same procedure was repeated separately for the psychosocial (eight models) and environmental (eight models) variables. Second, socio-demographic variables, psychosocial variables, and environmental variables for which at least a trend towards a significant relationship (p<0.10) was observed in the previous step were included in one final model for each dependent variable (eight models). Consequently, the final models for the different dependent variables might include different socio-demographic, psychosocial and environmental variables. Furthermore, for each dependent variable, different socio-demographic, psychosocial and environmental variables might be included in the zero-inflated model compared to the negative binomial model. Only the results of the eight final models are reported in the results section and the tables. Self-efficacy was only included in the models for walking and cycling as this was only assessed for active transport. Concerning public transport, the four variables representing social modelling were included separately due to low internal consistency (Cronbach’s alpha<0.6). Distance to school and facilities at school were only included in the models for transport to school. To examine the correlates of transport to school, only participants living within a feasible active commuting distance of eight kilometres from school were included in analyses (n = 306) [21]. P-values < 0.05 were considered statistically significant.

## Results

### Sample characteristics

After exclusion of participants showing unrealistic data (i.e. 1000 minutes cycling/day) on nearly all items (n = 2) and participants younger than 16 years or older than 19 years (n = 49), 562 participants (562/1145 = 49.1%) were included in the study.

Table 2 presents socio-demographic characteristics, general transport data and data on transport to school and other destinations. In total, 54.6% of the sample was female, mean age was 17.8 (0.7) years and 71.6% was a high SES adolescent. Furthermore, 73.1% of the sample lived in a rural area.

**Table 2:** Descriptive characteristics of the sample (%, Mean (SD)).

**Socio-demographic characteristics**	
Gender (% female)	54.6
Age (yrs)	17.8 (0.7)
BMI (kg/m^2^)	21.6 (3.0)
Nationality (% Belgian)	96.1
Living situation (% living with their (grand)parents)	95.6
Living environment (% living in rural area)	73.1
*Socio-economic status (SES)*	
low SES (% no parent has a Bachelor’s degree or higher)	28.4
high SES (% at least one parent has a Bachelor’s degree or higher)	71.6
*Grade*	
penultimate year of secondary school (%)	47.7
last year of secondary school (%)	52.3
*Education*	
General studies (%)	49.8
Technical studies (%)	29.9
Vocational studies (%)	20.2
**Transport in general**	
*Car/motorcycle*	
Driving license (%)	12.6
Ownership (%)	12.1
Sharing and/or borrowing capability (%)	49.7
*Moped*	
Driving license (%)	8.0
Ownership (%)	8.4
Sharing and/or borrowing capability (%)	2.3
*Bicycle*	
Ownership (%)	93.4
Sharing and/or borrowing capability (%)	2.3
Ownership public transport pass (%)	47.0
Ownership bicycle sharing schemes pass (%)	0.5
**Transport to school**	
Participants who walked (%)	21.3
Amount walking (minutes/week)	69 (53)
Participants who cycled (%)	48.8
Amount cycling (minutes/week)	131 (98)
Among participants who made use of public transport (%)	40.4
Amount public transport use (minutes/week)	249 (211)
Among participants who made use of passive transport (%)	24.6
Amount passive transport use (minutes/week)	113 (139)
**Transport to other destinations**	
Participants who walked (%)	39.4
Amount walking (minutes/week)	106 (158)
Participants who cycled (%)	51.7
Amount cycling (minutes/week)	125 (124)
Participants who made use of public transport (%)	38.1
Amount public transport use (minutes/week)	209 (195)
Participants who made use of passive transport (%)	50.0
Amount passive transport use (minutes/week)	120 (128)

The participants that were included in the final sample did not differ significantly from those participants who did not complete the questionnaire entirely (n = 505) with regard to gender (54.4% female; p = 0.932) and SES (71.4% high SES; p = 0.951). However, participants who did not complete the questionnaire were on average younger (17.2 (0.9) years; p < 0.001).

Mean scores of the psychosocial and environmental variables are shown in Table 1.

### Correlates of walking

Table 3 presents associations of psychosocial and environmental variables with walking. After controlling for socio-demographic variables, the logit model shows that older adolescents perceiving more social modelling for active transport had 29% lower odds of non-participation in walking to school. In other words, older adolescents perceiving more social modelling for active transport were more likely to walk to school. The negative binomial model shows that among older adolescents who walked to school in the last week, those with a one-unit higher social norm towards active transport walked 15% minutes less to school.

**Table 3:** Associations of psychosocial and environmental variables with walking.

	School		Other destinations	
	Logit model: OR of being non-participant^a^ (95% CI)	Negative binomial model: min/week (95% CI)	Logit model: OR of being non-participant^b^ (95% CI)	Negative binomial model: min/week (95% CI)
**Socio-demographic**				
SES (ref: low)				0.60 (0.45, 0.81)***
education (ref: vocational)	1.72 (0.97, 3.05)			
**Psychosocial**				
self-efficacy			1.13 (0.91, 1.40)	1.22 (1.02, 1.45)*
social norm	1.22 (0.99, 1.50)	0.85 (0.76, 0.95)**	1.18 (0.99, 1.40)	
social modelling	0.71 (0.55, 0.91)**		0.81 (0.66, 1.01)	
social support				1.09 (0.91, 1.29)
perceived benefits				0.92 (0.71, 1.19)
**Environmental**				
residential density			0.79 (0.65, 0.96)*	
land use mix access				0.88 (0.68, 1.13)
walking and cycling facilities	1.40 (0.86, 2.28)			
aesthetics			1.29 (0.91, 1.81)	
facilities at school	2.60 (0.82, 8.26)	0.57 (0.31, 1.05)		
distance	0.99 (0.97, 1.01)			

OR = odds ratio; CI = confidence interval;

* p<0.05,

** p<0.01,

*** p<0.001.

^a^ OR of being non-participant in walking to school;

^b^ OR of being non-participant in walking to other destinations

Socio-demographic variables, psychosocial variables, and environmental variables for which at least a trend towards a significant relationship (p<0.10) was observed in the first step were included in this final model.

ZINB models evaluate the correlates of the odds of non-participation in walking to school or to other destinations (logit model). Simultaneously, among participants who did walk to school or to other destinations, ZINB models evaluate the correlates of weekly minutes walking to school or to other destinations (negative binomial model). Negative binomial model parameters represent the proportional increase in minutes/week walking to school or to other destinations with a one-unit increase in the predictor.

For walking to other destinations, the logit model shows that living in a densely built neighbourhood was associated with 21% lower odds of non-participation in walking to other destinations. Hence, older adolescents living in densely built neighbourhoods were more likely to walk to other destinations. The negative binomial model shows that among older adolescents who walked to other destinations in the last week, those with a one-unit higher self-efficacy towards active transport walked 22% minutes more to other destinations.

### Correlates of cycling

Table 4 presents associations of psychosocial and environmental variables with cycling. In summary, older adolescents with higher self-efficacy towards active transport and those with a higher social norm towards active transport were more likely to cycle to school. Among older adolescents who cycled to school in the last seven days, an increase of 1 km in distance to school was related to 5% minutes more cycling to school.

**Table 4:** Associations of psychosocial and environmental variables with cycling.

	School		Other destinations	
	Logit model: OR of being non-participant^a^ (95% CI)	Negative binomial model: min/week (95% CI)	Logit model: OR of being non-participant^b^ (95% CI)	Negative binomial model: min/week (95% CI)
**Socio-demographic**				
gender (ref: female)			0.66 (0.40, 1.08)	1.05 (0.85, 1.30)
age		1.07 (0.92, 1.23)	1.32 (0.92, 1.88)	
BMI	0.99 (0.90, 1.08)			
SES (ref: low)	0.59 (0.31, 1.09)		0.60 (0.35, 1.02)	
education (ref: vocational)	0.52 (0.18, 1.49)		1.10 (0.48, 2.54)	
**Psychosocial**				
self-efficacy	0.27 (0.18, 0.42)***		0.42 (0.30, 0.60)***	1.48 (1.26, 1.72)***
social norm	0.64 (0.50, 0.82)***		0.62 (0.51, 0.77)***	
perceived benefits			0.90 (0.61, 1.32)	
perceived barriers	1.63 (0.99, 2.67)		1.20 (0.79, 1.83)	
**Environmental**				
residential density	1.22 (0.88, 1.69)		1.24 (0.94, 1.62)	0.86 (0.76, 0.97)*
land use mix diversity			1.29 (0.94, 1.78)	
land use mix access	0.75 (0.39, 1.46)		0.64 (0.36, 1.13)	
street connectivity	0.70 (0.39, 1.27)			
walking and cycling facilities		1.06 (0.82, 1.36)		
safety from crime	0.77 (0.50, 1.18)		0.87 (0.60, 1.25)	0.75 (0.63, 0.89)**
distance		1.05 (1.02, 1.07)***		

OR = odds ratio; CI = confidence interval;

* p<0.05,

** p<0.01,

*** p<0.001.

^a^ OR of being non-participant in cycling to school;

^b^ OR of being non-participant in cycling to other destinations

Socio-demographic variables, psychosocial variables, and environmental variables for which at least a trend towards a significant relationship (p<0.10) was observed in the first step were included in this final model.

ZINB models evaluate the correlates of the odds of non-participation in cycling to school or to other destinations (logit model). Simultaneously, among participants who did cycle to school or to other destinations, ZINB models evaluate the correlates of weekly minutes cycling to school or to other destinations (negative binomial model). Negative binomial model parameters represent the proportional increase in minutes/week cycling to school or to other destinations with a one-unit increase in the predictor.

Regarding cycling to other destinations, adolescents with higher self-efficacy towards active transport and those with a higher social norm towards active transport were more likely to cycle. Among older adolescents who cycled to other destinations in the last seven days, a one-unit increase in self-efficacy towards active transport was related to 48% minutes more cycling to other destinations. On the other hand, a one-unit increase in residential density and in perceived safety from crime was related to 14% and 25% minutes less cycling to other destinations, respectively.

### Correlates of public transport

Table 5 presents associations of psychosocial and environmental variables with public transport. In summary, older adolescents having a higher social norm towards public transport, those perceiving more social modelling from brothers/sister and from friends, those perceiving more social support regarding public transport, those perceiving a lower land use mix access and those living further from school were more likely to make use of public transport to school. Among older adolescents who went to school by public transport within the last week, a one-unit increase in perceived facilities at school in favour of walking and cycling, and an increase of 1 km in distance to school was related to 75% and 4% minutes more public transport to school, respectively.

**Table 5:** Associations of psychosocial and environmental variables with public transport.

	School		Other destinations	
	Logit model: OR of being non-participant^a^ (95% CI)	Negative binomial model: min/week (95% CI)	Logit model: OR of being non-participant^b^ (95% CI)	Negative binomial model: min/week (95% CI)
**Socio-demographic**				
gender (ref: female)				1.27 (0.95, 1.68)
age			0.81 (0.57, 1.14)	
BMI		1.04 (1.01, 1.08)**		
SES (ref: low)			1.47 (0.87, 2.48)	0.74 (0.55, 1.00)*
education (ref: vocational)	1.68 (0.92, 3.09)		2.16 (1.00, 4.64)*	0.72 (0.51, 1.01)
**Psychosocial**				
social norm	0.58 (0.45, 0.75)***	1.08 (0.98, 1.20)	0.66 (0.51, 0.86)**	1.04 (0.91, 1.18)
social modelling				
partner			1.02 (0.88, 1.18)	0.94 (0.86, 1.02)
parents				1.07 (0.95, 1.21)
brothers/sisters	0.86 (0.75, 0.99)*			1.08 (0.99, 1.18)
friends	0.76 (0.62, 0.93)**		0.76 (0.62, 0.94)*	
social support	0.61 (0.42, 0.87)**		0.60 (0.42, 0.87)**	
**Environmental**				
land use mix access	2.31 (1.40, 3.81)**		2.15 (1.33, 3.45)**	
street connectivity	1.62 (0.97, 2.70)			
safety from traffic		1.01 (0.76, 1.34)		
safety from crime		0.97 (0.83, 1.12)		
facilities at school		1.75 (1.07, 2.86)*		
distance	0.93 (0.90, 0.96)***	1.04 (1.03, 1.05)***		

OR = odds ratio; CI = confidence interval;

* p<0.05,

** p<0.01,

*** p<0.001.

^a^ OR of being non-participant in public transport to school;

^b^ OR of being non-participant in public transport to other destinations

Socio-demographic variables, psychosocial variables, and environmental variables for which at least a trend towards a significant relationship (p<0.10) was observed in the first step were included in this final model.

ZINB models evaluate the correlates of the odds of non-participation in public transport to school or to other destinations (logit model). Simultaneously, among participants who did use public transport to go to school or to other destinations, ZINB models evaluate the correlates of weekly minutes public transport to school or to other destinations (negative binomial model). Negative binomial model parameters represent the proportional increase in minutes/week public transport to school or to other destinations with a one-unit increase in the predictor.

Regarding public transport to other destinations, older adolescents perceiving a higher social norm towards public transport, those perceiving more social modelling from friends, those perceiving more social support regarding public transport, and those perceiving lower land use mix access were more likely to make use of public transport to travel to other destinations. Among those who used public transport to go to other destinations within the last week, none of the variables included in the negative binomial model were significantly associated with the amount of minutes public transport.

### Correlates of passive transport

Table 6 presents associations of psychosocial and environmental variables with passive transport. In summary, older adolescents perceiving more social support regarding passive transport, those with more perceived benefits regarding passive transport, and those perceiving less facilities at school in favour of walking and cycling were more likely to commute passively to school. Among older adolescents who commuted passively to school within the last week, an increase of 1 km in distance to school was related to 5% minutes more passive commuting to school.

**Table 6:** Associations of psychosocial and environmental variables with passive transport.

	School		Other destinations	
	Logit model: OR of being non-participant^a^ (95% CI)	Negative binomial model: min/week (95% CI)	Logit model: OR of being non-participant^b^ (95% CI)	Negative binomial model: min/week (95% CI)
**Socio-demographic**				
gender (ref: female)	1.87 (1.15, 3.03)*		1.78 (1.18, 2.69)**	
SES (ref: low)		0.93 (0.62, 1.38)	0.40 (0.25, 0.64)***	
education (ref: vocational)				0.50 (0.36, 0.70)***
**Psychosocial**				
social modelling	0.94 (0.73, 1.21)		0.77 (0.62, 0.95)*	
social support	0.59 (0.43, 0.81)**		0.52 (0.39, 0.69)***	1.18 (1.02, 1.36)*
perceived benefits	0.64 (0.48, 0.85)**		0.75 (0.59, 0.94)*	
perceived barriers	1.27 (0.97, 1.66)			0.84 (0.74, 0.97)*
**Environmental**				
residential density			1.12 (0.90, 1.38)	
land use mix access	1.63 (1.00, 2.68)		1.19 (0.79, 1.78)	0.94 (0.74, 1.20)
walking and cycling facilities				0.80 (0.62, 1.04)
aesthetics			0.78 (0.54, 1.13)	
safety from traffic				1.25 (0.91, 1.71)
safety from crime		0.91 (0.70, 1.19)		0.86 (0.73, 1.02)
facilities at school	6.35 (1.87, 21.48)**			
distance	1.00 (0.97, 1.02)	1.05 (1.02, 1.07)***		

OR = odds ratio; CI = confidence interval;

* p<0.05,

** p<0.01,

*** p<0.001.

^a^ OR of being non-participant in passive transport to school;

^b^ OR of being non-participant in passive transport to other destinations

Socio-demographic variables, psychosocial variables, and environmental variables for which at least a trend towards a significant relationship (p<0.10) was observed in the first step were included in this final model.

ZINB models evaluate the correlates of the odds of non-participation in passive transport to school or to other destinations (logit model). Simultaneously, among participants who did commute passively to school or to other destinations, ZINB models evaluate the correlates of weekly minutes passive transport to school or to other destinations (negative binomial model). Negative binomial model parameters represent the proportional increase in minutes/week passive transport to school or to other destinations with a one-unit increase in the predictor.

Regarding passive transport to other destinations, older adolescents perceiving more social modelling for passive transport, those perceiving more social support towards passive transport and those with more perceived benefits regarding passive transport were more likely to use passive transport. Among older adolescents who used passive transport to other destinations in the last seven days, a one-unit increase in social support regarding passive transport was associated with 18% minutes more passive transport to other destinations. Finally, a one-unit increase in perceived barriers regarding passive transport was associated with 16% minutes less passive commuting to other destinations.

Results of the eight final models are summarized in one table (Table 7) to provide a clear overview of significant psychosocial and environmental associations with the different transport modes.

**Table 7:** Overview of significant psychosocial and environmental associations with different transport modes.

	Walking		Cycling		Public transport		Passive transport	
	School	Other destinations	School	Other destinations	School	Other destinations	School	Other destinations
**Psychosocial**								
self-efficacy		+ ^(NB)^	+ ^(L)^	+ ^(L)^; + ^(NB)^				
social norm	- ^(NB)^		+ ^(L)^	+ ^(L)^	+ ^(L)^	+ ^(L)^		
social modelling	+ ^(L)^				+ ^(L)^	+ ^(L)^		+ ^(L)^
social support					+ ^(L)^	+ ^(L)^	+ ^(L)^	+ ^(L)^; + ^(NB)^
perceived benefits							+ ^(L)^	+ ^(L)^
perceived barriers								- ^(NB)^
**Environmental**								
residential density		+ ^(L)^		- ^(NB)^				
land use mix diversity								
land use mix access					- ^(L)^	- ^(L)^		
street connectivity								
walking and cycling facilities								
aesthetics								
perceived safety from traffic								
perceived safety from crime				- ^(NB)^				
facilities at school					+ ^(NB)^		- ^(L)^	
distance			+ ^(NB)^		+ ^(L)^; + ^(NB)^		+ ^(NB)^	

^(L)^ = logit model; ^(NB)^ = negative binomial model

+ ^(L)^ = higher odds of being participant; − ^(L)^ = lower odds of being participant; + ^(NB)^ = more minutes/week; − ^(NB)^ = less minutes/week

ZINB models evaluate the correlates of the odds of non-participation in a transport mode to school or to other destinations (logit model). Simultaneously, among participants who did use that transport mode to school or to other destinations, ZINB models evaluate the correlates of weekly minutes for that transport mode to school or to other destinations (negative binomial model). Negative binomial model parameters represent the proportional increase in minutes/week for that transport mode to school or to other destinations with a one-unit increase in the predictor.

## Discussion

This was the first study to investigate correlates of walking, cycling, public transport as well as passive transport over short distances in older adolescents, not only to school but also to other destinations. Although older adolescence is a critical period characterized by major life changes and the possibility to obtain a driving license, it is an overlooked age group regarding transport behaviour.

More social modelling of significant others for active transport was related to older adolescents being more likely to walk to school. To our knowledge, only one US study investigated parental walking for transportation in relation to adolescents’ active commuting to school (both walking and cycling) [19]. They did not find a significant association between social modelling of parents and adolescents’ active transport. However, previous Belgian studies found that general physical activity levels of family and/or friends [21,28] were positively related to older adolescents’ active transport to school and/or other destinations. In the present study, higher residential density of the neighbourhood was positively related to the odds of walking to other destinations. This indicates that a good accessibility of destinations in the neighbourhood can promote walking for transport. Previous studies among adolescents found that there is a positive relation between urbanisation or density of living environment and active transport to school, including both walking and cycling [19,20,26,44]. Nevertheless, in the current study, density of the neighbourhood was not significantly related to the odds of cycling probably because older adolescents can also reach destinations outside their neighbourhood by bicycle.

Regarding cycling, participants with higher self-efficacy towards active transport were more likely to cycle to school and other destinations. Furthermore, a higher self-efficacy resulted in more minutes cycling to other destinations among those who cycled within the last week. This is in line with a previous study among Belgian older adolescents in which higher self-efficacy (towards physical activity in general) was related to more active transport (including walking and cycling) to school and to other destinations [28]. However, in the present study, self-efficacy was not significantly associated with engaging in walking. Older adolescents seem to prefer walking as a transport mode only for very short distances [27]. It is possible that there are only very few barriers to overcome for these short walking trips compared to cycling (e.g. bad weather, sweating, carrying things). Furthermore, a higher social norm towards active transport resulted in older adolescents being more likely to cycle to school and to other destinations. A previous study among Belgian older adolescents did not find a significant relation between social norm and active transport to school [21]. Taking into account that cycling for transport has several health benefits [45], future interventions promoting active transport among older adolescents need to focus on increasing self-efficacy and social norm towards cycling within this age group. None of the environmental variables was significantly related to the odds of cycling.

To the best of our knowledge, no previous studies have quantitatively examined psychosocial and environmental correlates of public transport and passive transport among (older) adolescents. The present study revealed that a higher social norm, more social modelling of siblings and/or friends and more social support regarding public transport resulted in older adolescents being more likely to use public transport to school and to other destinations. In accordance, a qualitative study among British adolescents found that social interactions with friends and travelling together were important for adolescents to choose for public transport [30]. Regarding environmental variables, the present study indicated that a lower land use mix access resulted in older adolescents being more likely to use public transport to school and to other destinations. This can be explained by the fact that in neighbourhoods characterised by a lower land use mix access walking and cycling are not the most convenient transport modes to reach a destination and public transport might be a suitable alternative. Since it is difficult to promote active transport in neighbourhoods with low land use mix and public transport generally involves some walking or cycling [10,11], promoting public transport might be beneficial as it provides the opportunity to cover part of the distance actively.

Similar to the other transport modes, psychosocial factors seemed to be of major importance for passive transport. Older adolescents perceiving more social support and those with more perceived benefits towards passive transport were more likely to use passive transport to go to school and to other destinations. More social support also resulted in more minutes passive transport to other destinations among those who used passive transport within the last week. Those perceiving more social modelling for passive transport were more likely to use passive transport to other destinations. Furthermore, the present study revealed that older adolescents who used passive transport to go to school perceived less facilities at school in favour of walking or cycling. This indicates that schools also have a certain responsibility in the promotion of active transport by providing adequate facilities at school in favour of walking and cycling (e.g. adequate bicycle storage, showers, …), and by informing their students about the presence of these facilities.

Overall, psychosocial variables seemed to be more important than environmental variables across the four transport modes among older adolescents. Previous studies in adults also concluded that mainly personal and social factors were associated with cycling for transport [36,46]. Social norm, social modelling and social support were the most consistent psychosocial correlates. This indicates that interventions promoting active transport might benefit from also targeting significant others. In the present study it seemed that siblings and friends were the most important for older adolescents’ transport behaviour. Walking or cycling together with siblings or friends has the potential to increase social norm, social modelling and social support towards active transport. A previous study showed that parents still influence transport behaviour of adolescents despite their independent mobility and even have a bigger influence on adolescents’ transport behaviour than peers [13]. Nevertheless, in a qualitative study among Belgian older adolescents, participants declared that their friends have a considerable influence on their transport behaviour [27].

The fact that Flanders is a walking- and cycling-friendly region with adequate infrastructure and facilities to support walking and cycling for transport can explain the finding that psychosocial variables were more important than environmental variables in the present study. Furthermore, Flanders is also characterized by good geographical and climatological conditions for active transport. In accordance, a focus group study among Belgian older adolescents showed that the built environment did not influence their transport behaviour whereas social factors were of greater importance [27]. In countries where much more environmental barriers are present, both psychosocial and environmental variables might be important regarding transport behaviour among (older) adolescents. Nevertheless, even in Flanders, there is still room to improve infrastructure to support walking and cycling. Specifically with regard to children a safe environment with adequate walking and cycling infrastructure is essential in the promotion of active transport [47,48].

### Limitations and strengths

A first limitation of this study is that no causal relationships could be drawn due to the cross-sectional study design. Second, a self-reported questionnaire was used which could lead to participants’ over-/underestimating the use of questioned transport modes and distance to school. Future studies should consider including both objective (using for example GPS) and subjective measures of transport behaviour. Third, adolescents following general studies (higher SES) in the last two years of secondary school were over-represented compared to the total population of adolescents in Flanders during the school year 2012–2013 (49.1% versus 36.2%) [49]. Fourth, because of the walking- and cycling-friendly characteristics of Flanders (Belgium) [50,51], results cannot be generalised to less walking- and cycling-friendly countries/continents. Finally, results of the negative binomial model for walking (n = 52), public transport (n = 76) and passive transport (n = 79) to school need to be interpreted with caution. Post-hoc power analyses showed that a sample size of 103 participants was needed for each of the regression models. Because of the insufficient sample size for these specific models, it is possible that the present study did not detect an association between one of the dependent variables and the psychosocial and environmental factors that would have been detected with a larger sample.

A first strength of this study is the large sample size (n = 562). A second strength is the chosen target group since evidence on correlates of these transport modes in this age group is very limited. Third, psychosocial correlates were surveyed separately for active transport (instead of physical activity in general), for public transport and for passive transport. Fourth, due to the lack of knowledge about correlates of public and passive transport in older adolescents, these transport modes were included in the present study next to active transport, in contrast to previous studies. Fifth, correlates of the different transport modes were investigated for both transport to school and transport to other destinations. Finally, psychosocial as well as environmental variables were investigated simultaneously.

## Conclusions

The present study revealed a broad array of variables related to walking, cycling, public transport and passive transport. Overall, psychosocial variables seemed to be more important than environmental variables across the four transport modes among older adolescents. Social norm, social modelling and social support were the most consistent psychosocial factors among the four transport modes which indicates that it is important to target both older adolescents and their social environment in interventions promoting active transport. Walking or cycling together with siblings or friends has the potential to increase social norm, social modelling and social support towards active transport. Flanders’ good geographical and climatological conditions for active transport and adequate walking and cycling infrastructure can explain the finding that environmental variables were less important in the present study.

## Supporting Information

S1 Dataset Raw data obtained from the online questionnaire. (XLSX)
